# Ovarian cancer cells regulate their mitochondrial content and high mitochondrial content is associated with a poor prognosis

**DOI:** 10.1186/s12885-023-11667-8

**Published:** 2024-01-08

**Authors:** Jil Weigelt, Mariam Petrosyan, Leticia Oliveira-Ferrer, Barbara Schmalfeldt, Catharina Bartmann, Johannes Dietl, Christine Stürken, Udo Schumacher

**Affiliations:** 1 Institute of Anatomy and Experimental Morphology, University Cancer Center Hamburg, University Medical Center Hamburg-Eppendorf, Martinistrasse 52, 20246 Hamburg, Germany; 2 Department of Gynecology, University Medical Center Hamburg-Eppendorf, Martinistrasse 52, 20246 Hamburg, Germany; 3 Department of Obstetrics and Gynaecology, University of Wuerzburg, 97080 Würzburg, Germany; 4 Department of Medicine, Medical School Hamburg, University of Applied Sciences and Medical University, Am Kaiserkai 1, 20457 Hamburg, Germany; 5 Department of Medicine, Faculty of Science, Medical School of Berlin, Berlin, Germany

**Keywords:** Immunohistochemistry, Intraperitoneal metastases, Mitochondria, Ovarian cancer, Ovarian cancer prognosis, Ovarian cancer xenografts

## Abstract

Most cancer patients ultimately die from the consequences of distant metastases. As metastasis formation consumes energy mitochondria play an important role during this process as they are the most important cellular organelle to synthesise the energy rich substrate ATP, which provides the necessary energy to enable distant metastasis formation. However, mitochondria are also important for the execution of apoptosis, a process which limits metastasis formation. We therefore wanted to investigate the mitochondrial content in ovarian cancer cells and link its presence to the patient’s prognosis in order to analyse which of the two opposing functions of mitochondria dominates during the malignant progression of ovarian cancer. Monoclonal antibodies directed against different mitochondrial specific proteins, namely heat shock proteins 60 (HSP60), fumarase and succinic dehydrogenase, were used in immunohistochemistry in preliminary experiments to identify the antibody most suited to detect mitochondria in ovarian cancer cells in clinical tissue samples. The clearest staining pattern, which even delineated individual mitochondria, was seen with the anti-HSP60 antibody, which was used for the subsequent clinical study staining primary ovarian cancers (*n* = 155), borderline tumours (*n* = 24) and recurrent ovarian cancers (*n* = 26). The staining results were semi-quantitatively scored into three groups according to their mitochondrial content: low (*n* = 26), intermediate (*n* = 50) and high (*n* = 84). Survival analysis showed that high mitochondrial content correlated with a statistically significant overall reduced survival rate In addition to the clinical tissue samples, mitochondrial content was analysed in ovarian cancer cells grown in vitro (cell lines: OVCAR8, SKOV3, OVCAR3 and COV644) and in vivo in severe combined immunodeficiency (SCID) mice.

In in vivo grown SKOV3 and OVCAR8 cells, the number of mitochondria positive cells was markedly down-regulated compared to the in vitro grown cells indicating that mitochondrial number is subject to regulatory processes. As high mitochondrial content is associated with a poor prognosis, the provision of high energy substrates by the mitochondria seems to be more important for metastasis formation than the inhibition of apoptotic cell death, which is also mediated by mitochondria. In vivo and in vitro grown human ovarian cancer cells showed that the mitochondrial content is highly adaptable to the growth condition of the cancer cells.

## Introduction

Despite the fact that recently considerable advances in cancer therapy have been made, cancer is still the second leading cause of death worldwide after cardiovascular diseases [1]. For many cancers, this high mortality rate is due to the formation of distant metastases [2] and in ovarian cancer in particular metastasis formation takes the rare route of intraperitoneal metastasis formation, which is also associated with a poor overall prognosis [3]. A plethora of drugs and treatment regimens have been developed, but in most cases none have proved to be superior in long-term. Although platinum-based chemotherapy is the gold standard treatment, there is often a high risk of platinum resistance [4, 5]. One of the reasons that intraperitoneal ovarian cancer may be so difficult to treat is that ovarian cancer cells show considerable plasticity [6] and undergo epithelial mesenchymal transition, which is associated with chemotherapy resistance [7, 8].

Chemotherapy-induced apoptosis is often mediated by mitochondrial apoptotic pathways therefore they represent attractive targets for chemotherapeutic drugs [9]. Hence, by down-regulating their mitochondrial numbers, cancer cells can escape the chemotherapeutic attack. However, the primary function of mitochondria is the synthesis of adenosine triphosphate (ATP), an energy rich substrate which is essential for cell proliferation and proper cellular function [10]. These functions also include the synthesis of intermediary metabolites which serve as building blocks of macromolecules such as nucleosides for DNA synthesis and hence targeting mitochondria by specific drugs has become an emerging field of drug development [11–13]. This important metabolic function of mitochondria is reflected in the long-established “Warburg hypothesis” to explain tumour cell progression, which defines excess lactate production in the presence of oxygen as mitochondrial dysfunction [14]. Today, however, it is more likely that the increased aerobic glycolysis recorded by Warburg represents an adaptation of tumour cells to hypoxia, which becomes more prominent as cancer progresses because the role of mitochondria is essential for tumour cells [15, 16].

Thus, mitochondria have two opposing roles in malignant progression; on the one hand, they are considered important components to produce high energy substrates, which are particularly important for providing energy for the metastatic spread of cancer cells. However, they are also important for the cytosolic release of pro-apoptotic proteins, which leads to the limitation of metastatic spread [17, 18].

As it is not clear which of the two effects of mitochondria predominates during malignant progression in ovarian cancer, that is, whether the presence of mitochondria is more advantageous for intraperitoneal spread due to the synthesis of the energy rich substrate ATP, or whether mitochondrial down-regulation prevents the apoptosis of cancer cells thereby leading to a survival advantage, we investigated the presence of mitochondria in ovarian cancer cells and correlated their presence with the patients’ survival.

## Materials and methods

### Patients

Mitochondrial density was analysed in 24 borderline tumours, 155 primary ovarian cancers, and 26 recurrent ovarian cancer samples. Formalin-fixed paraffin-embedded (FFPE) samples were obtained from the files of the University Medical Center Hamburg-Eppendorf. These were collected during surgeries from patients with primary and recurrent disease treated between 2016 and 2020. The FFPE samples from the Würzburg University Hospital were collected between 1990 and 1997. The cohorts contain tissue from different locations such as the fallopian tube, the ovary itself and metastatic sites (omentum and peritoneal wall). The histological and clinical data of both collectives are shown in Table 1.

**Table 1:** Descriptive statistics of the cohort

***n* = 205**		**Hamburg**		**Wurzburg**
		***n* = 139**		***n* = 66**
		Patient number		Patient number
**Borderline tumour**		17 (12%)		7 (11%)
**Recurrent ovarian cancer**		20 (14%)		6 (9%)
**Primary ovarian cancer**		102^a^ (74%)		53 (80%)
Age at diagnosis (y)	Mean (median)	59.3 y (60 y)		56.5 y (58.5 y)
Histological type	Serous papillary	93 (67%)		62 (94%)
	Endometrioid	2 (1%)		1 (2%)
	Clear cell	1 (0,7%)		1 (2%)
	Müllerian mucinous	5 (4%)		2 (3%)
FIGO state	FIGO I-II	2 (1%)		30 (45%)
	FIGO IIIA-IIIB	6 (4%)		10 (15%)
	FIGO IIIC	69 (50%)		22 (33%)
	FIGO IV	17 (12%)		4 (6%)
Nodal involvement	Negative	14 (10%)		
	Positive	54 (39%)		
Grading	Low grading	3 (2%)		19 (29%)
	High grading	85 (61%)		47 (71%)
Distant metastasis	Negative	72 (52%)		62 (94%)
	Positive	20 (14%)		4 (6%)
Tumour residuum after surgery	Not macroscopically visible	53 (38%)		33 (50%)
	< 1cm^3^	23 (17%)		5 (8%)
	> 1cm^3^	25 (18%)		28 (42%)
Adjuvant chemotherapy	Carboplatin/Paclitaxel (Taxol)	97 (70%)	Cyclophosphamide/Carboplatin	10 (15%)
	Bevacizumab	61 (43%)	Carboplatin mono	3 (5%)
			Cyclophosphamide/Cisplatin	36 (55%)
			None	17 (26%)
Recurrence	Yes	70 (50%)		14 (21%)
	No	32 (23%)		35 (53%)
			Inoperable	17 (26%)
Recurrence-free survival (month)	Mean (median)	23.88 (21)		30.07 (22)
Overall survival (month)	Mean (median)	36 (33.5)		39.09 (19)

^a^missing values to *n* = 102; unknown

All patients of the Hamburg cohort provided written informed consent prior to their biological material and clinical records being accessed according to the guidelines of the Review and Ethics Committee (#190504 and PV6012) of the University Medical Center Hamburg-Eppendorf [19]. The histological sections have been used in a previous study where the regulatory approvals are described [20]. Their clinical data were obtained from a detailed institutional database containing information on surgical, therapeutic and clinicopathological procedures [19]. The clinical data of the Würzburg cohort as well as the associated biosamples were used in anonymised form. This complies with ethical and current legal requirements requested by the Ethics Committee of the University of Würzburg. This study is also fully compliant with the Declaration of Helsinki.

### Cell lines and cell culture

Human ovarian cancer cell lines OVCAR8 (Medium uses for OVCAR8: Roswell Park Memorial Institute (RPMI-1640) medium (Gibco)), SKOV3 (Medium used for SKOV3: McCoy’s 5a Medium Modified (Gibco)), OVCAR3 (Medium uses for OVCAR3: Roswell Park Memorial Institute (RPMI-1640) medium (Gibco)) and COV644 (Medium used for COV644: DMEM (Gibco)) were analysed. All four cell lines grew as adherent cells. The medium was supplemented with 10% (SKOV3, OVCAR8, COV644) or 20% (OVCAR3) fetal calf serum (FCS) and 2 mM L-glutamine, 100 units/mL penicillin, 100 g/mL streptomycin (GibcoTM, Waltham, MA, USA) as seen in Table 2. Cells were maintained in a humidified atmosphere of 95% air and 5% CO_2_ at 37 °C. If confluence reached 70–80%, cells were passaged to expand them. Subsequently, immunohistochemical detection was performed and evaluated from in vitro cultured SKOV3, OVCAR3, and COV644 ovarian cancer cells fixed in formalin and embedded in wax (FFPE) as described below.

**Table 2:** Cell lines

Cell line	Type	Tissue	Source	Medium
SKOV3	Serous cystadenocarcinoma	Ascites	ATCC® HTB77™	McCoy’s 5A + 10% FCS and 1% P/S, 2 mM L-Glu
OVCAR3	Poorly differentiated papillary Adenocarcinoma	Ascites	D. Thormeyer, Fachklinik Hornheide	RPMI + 20% FCS and 1% P/S, 2 mM L-Glu
OVCAR8	Adenocarcinoma	Ovary	V. Aßmann, UKE Hamburg	RPMI + 10% FCS and 1% P/S, 2 mM L-Glu
COV644	Mucinous ovarian carcinoma	Solid tumour, ovary	ECACC 07071908	DMEM + 10% FCS and 1% P/S, 2 mM L-Glu

### Xenograft samples and cells in agar

FFPE sections of human ovarian cancer cell lines OVCAR3 and SKOV3 grown as intraperitoneal tumours in SCID mice from previously published experiments were drawn from the files of the Institute of Anatomy and Experimental Morphology and processed in the same way as the clinical samples [19, 21–23]. In addition, in vitro grown ovarian cancer cells were fixed in formalin and embedded in agar in previous experiment of out institute (for cell lines used see section below) [24]. These agar embedded cell pellets were processed in the same way as the tumour tissue samples grown in scid mice. By this approach, differences in the number of mitochondria noted between in vivo and in vitro grown cells cannot be attributed to methodological differences in tissue processing.

### Immunohistochemistry (IHC)

Four µm thick FFPE sections were deparaffinised in two changes of xylene (5 min each), rehydrated in a series of graded ethanol (100%, 96%, 70% and 50% for 5 min each) and finally washed for two min in distilled water. For antigen retrieval, sections were pre-treated with a steamer (Dako Cytomation, Carpinteria, CA, USA) for 20 min at 100 °C in the case of anti-heat shock protein (HSP) 60 (NBP1-77,397, 1 mg/mL, Novus Biologicals, Colorado, USA), 99 °C for 10 min for anti-fumarase (NBP1-31,336, 0,9 mg/mL, Novus Biologicals, Colorado, USA) or 125 °C for 4 min in the case of anti-succinic dehydrogenase antibody (SDH; HPA041981, Atlas Antibodies, Bromma, Sweden) in a 1:10 dilution of epitope retrieval solution (#S1699, pH 6,0 Dako, Carpinteria, CA, USA). After cooling, the sections were rinsed twice with Tris-buffered saline/0.1% Tween20 (TBS-T) and once with TBS (pH 7.6) for 5 min each. The performance of the following steps took place in a humid chamber. The incubation was done with either anti-HSP 60 antibody (#NBP1-77397, Novus Biologicals), diluted 1:400 in Antibody Diluent (#S0809, Dako Carpinteria, CA, USA), or anti-fumarase antibody (#NBP1-31336, Novus Biologicals) diluted 1:1000 in Antibody Diluent or anti-SDH antibody (#HPA041981, Atlas) diluted 1:50 in Antibody Diluent at room temperature (RT) for 60 min. Rabbit poly IgG antibody (#ab37415, abcam, Berlin, Germany) diluted 1:2000 (HSP60), 1:5556 (fumarase) or 1:834 (SDH) in Antibody Diluent was used as an isotype control. After incubation, the sections were rinsed twice with TBS-T and once with TBS for 5 min each. Next, the sections were incubated with a secondary biotin-conjugated swine-α rabbit antibody (#E0353, Dako, Carpinteria, CA, USA) diluted 1:200 in TBS for 30 min at RT. This was followed by another washing step with TBS and TBS-T for 5 min each.

Subsequently, the sections were incubated with Vectastain® ABC-AP kit (#AK5000, Linaris, Dossenheim, Germany) according to the manufacturer’s recommendation at RT for 30 min followed by washing again in TBS-T and TBS as described above. The sections were incubated with the Permanent Red solution (#ZUC001-125, Zytomed Systems GmbH, Berlin, Germany) for 10 min (HSP60), for 20 min (Fumarase) and for 12 min (SDHA), respectively, to visualise the alkaline phosphatase enzyme activity. Subsequently, counterstaining was performed with hemalumn for 8 s and the sections were rinsed under running tap water for 5 min and in Aqua dest for 2 min.

FFPE embedded human kidney served as a positive control as mitochondria are abundantly present in the proximal convoluted tubule of the kidney.

Finally, the slides were dehydrated in a series of graded ethanol (70% for 15 s, 96% and 100% for 5 min each) and three changes of xylene (5 min each) and coverslipped with Eukitt® mounting medium (#03989, Sigma-Aldrich, Taufkirchen, Germany).

### Microscopy and image analysis

The sections were visually analysed using a ZEISS Axiophot 2 microscope (Carl Zeiss, Jena, Germany). After the initial visual control, the sections were digitised using a ZEISS AXIO Scan Z1 slide scanner equipped with a ZEISS EC Plan-Neofluar 20 × **/**0.50 pole M27 objective (Carl Zeiss, Jena, Germany) and a Hitachi HV-F20SCL 1600 × 1200 pixels camera (Hitachi Kokusai Electric America Ltd., New York, NY, USA). ZEISS ZEN 2.3 software (Carl Zeiss, Jena, Germany) was used for image acquisition. The netScope Viewer software (Net-Base Software, Freiburg, Germany) was used for further image processing.

The staining results were semi-quantitatively scored into three groups according to their mitochondrial content: low, intermediate, and high.

### Flow cytometry with MitoTracker Green

All four cell lines (see Table 2; at ~ 80% confluency) were trypsinised and afterwards centrifuged at 1500 rpm for 5 min. The supernatant was discarded, and the remaining pellets were re-suspended in 100 µL of pre-warmed (37℃) 60 nM MitoTracker™ Green FM (Thermo Fisher Scientific, USA) prepared according to the manufacturer’s instructions.

The cells were then incubated for 30 min under standard cell culture conditions (at 37℃ in 5% CO_2_, 95% humidity). The incubation was followed by a washing step, using flow cytometry buffer (1% BSA, 0.05% NaN3 in Dulbecco's phosphate-buffered saline). After centrifugation, the cell pellets were re-suspended in flow cytometry buffer, and the fluorescence was measured using the BD FACSCanto II Flow Cytometer. Propidium iodide (PI) (1 µg/mL) was used to stain the dead cells. The analysis of the flow cytometry was done using FlowLogic software (Inivai, Mentone Victoria, Australia).

### Statistical analyses

Statistical analyses were performed using Microsoft Excel version 16.47 (One Microsoft Way, Redmond, WA, USA). IHC sections were semi-quantitatively classified into low ( +), middle (+ +) or high (+ + +) mitochondrial content. The possible correlation between this quantification and histology (borderline, primary ovarian cancer and recurrent ovarian cancer) and clinic-pathological factors (Fédération Internationale de Gynécologie et d'Obstétrique (FIGO)-Classification, tumour stage, nodal involvement, histological subtype, grading and postoperative residual tumour) was analysed. Survival curves were analysed using GraphPad PRISM 9 (GraphPad Software, San Diego, USA). In addition, we conducted a multivariate analysis to include other clinical parameters. This was done with SPSS software Version 25 (IBM SPSS Statistics, Armonk, NY, USA). The correlations between mitochondrial content, survival and clinicopathologic factors (FIGO and residual postoperative tumor) were analysed using a multivariate Cox proportional hazards regression model. Probability values (*p* values) less than 0.05 were considered statistically significant.

## Results

### Mitochondrial content and distribution in different ovarian carcinomas

**Fig. 1:**
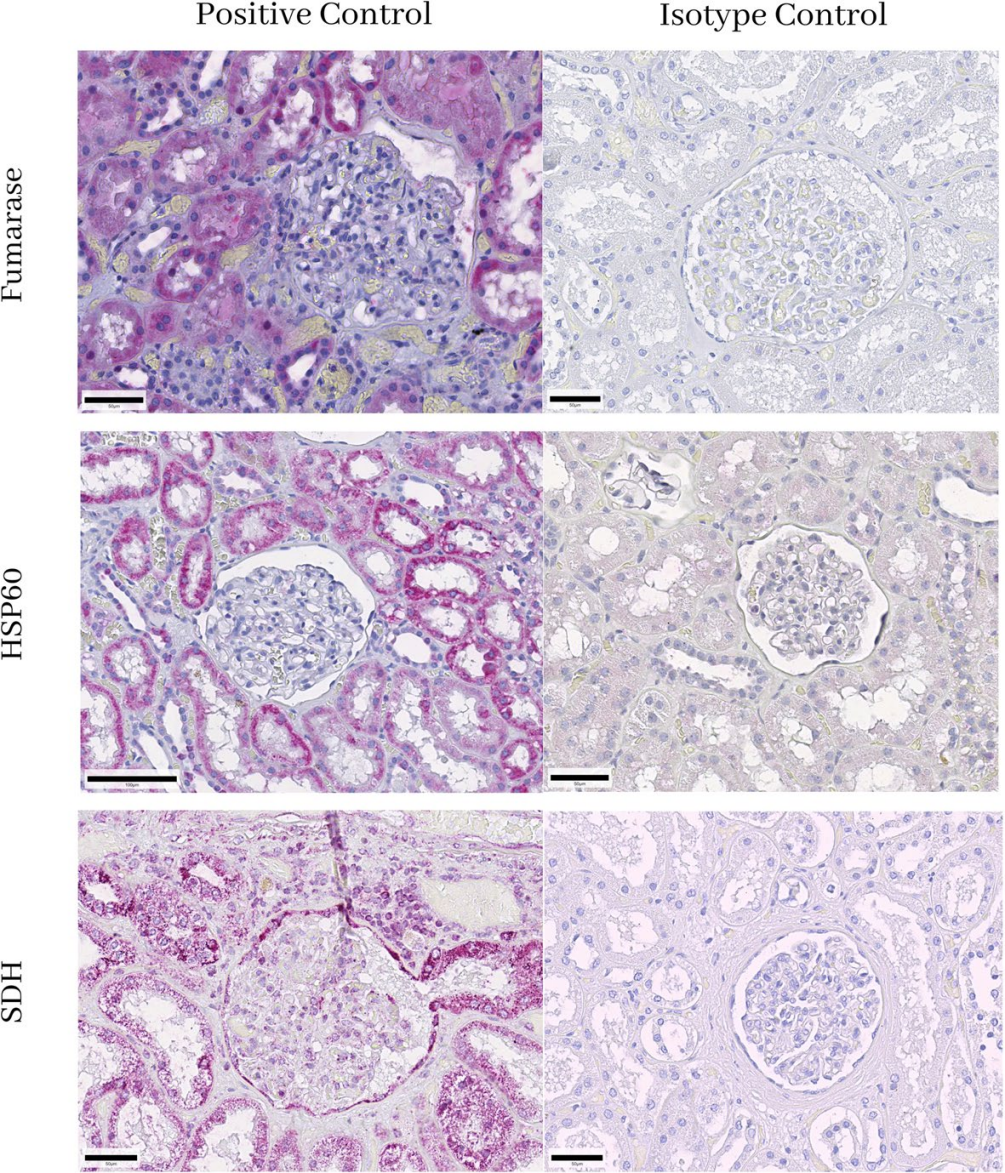
Fumarase, heat shock protein-60 (HSP60) and succinic dehydrogenase (SDH) immunoreactivity in the human kidney. All three antibodies labelled mitochondria in the proximal convoluted tubule, in the glomerulus positive immunoreactivity was only found to a very limited extend and individual mitochondria could only be detected at very high-power magnification (data not shown). Isotype controls did not show any immunoreactivity. The scale bar represents 50 µm on the left side (100 µm at HSP60) and 50 µm on the right side

**Fig. 2:**
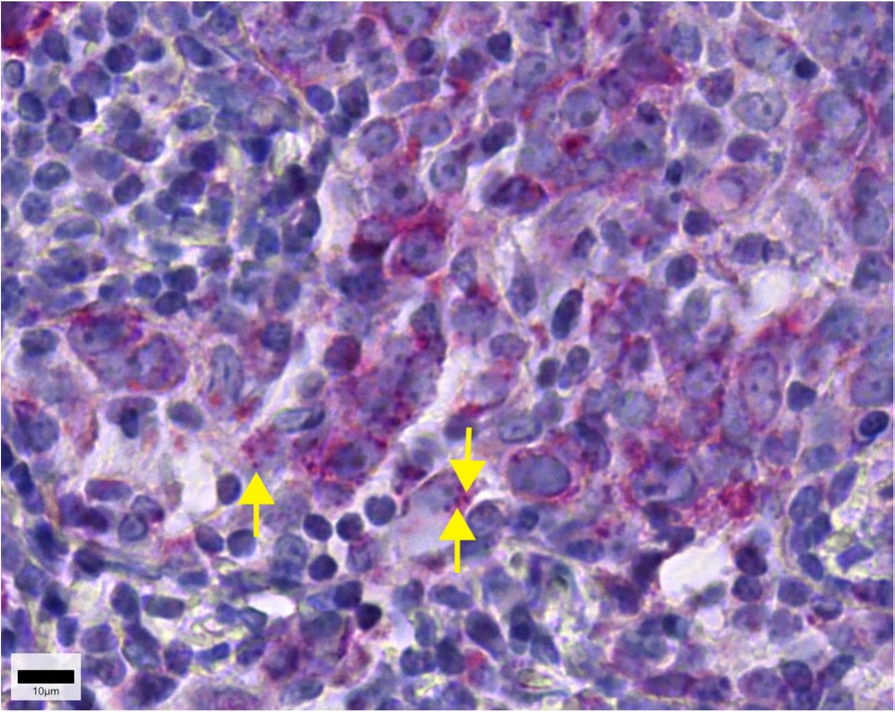
Serous papillary ovarian cancer treated with HSP60 immunohistochemistry. Note that individual mitochondria can be recognized (arrows)

**Fig. 3:**
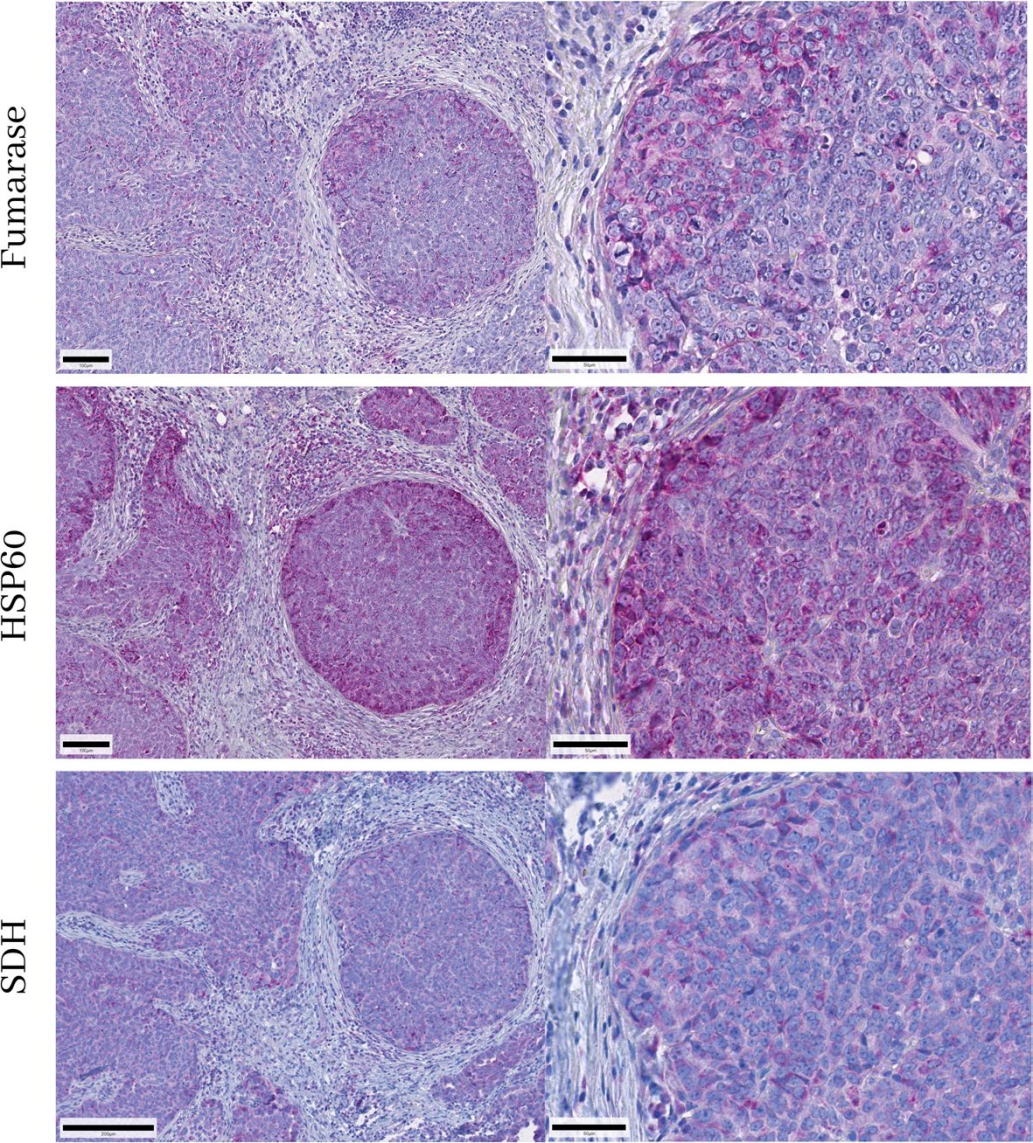
Serous papillary ovarian cancer with intermediate mitochondrial content. Note that the absence of cells containing mitochondria is common to all three antibodies. The most intensive staining was observed in HSP60 immunohistochemistry. The scale bar represents 100 µm on the left side (200 µm at SDH) and 50 µm on the right side

First, the presence or absence of immunohistochemically detectable mitochondria was validated in human kidneys and in different clinical samples of ovarian cancers using three monoclonal antibodies directed against different mitochondrial antigens, namely HSP60, fumarase and SDHA. All three antibodies detected mitochondria in the proximal tubule of the kidney and the isotype controls were negative (Fig. 1). Individual mitochondria could be identified using the HSP-60 monoclonal antibody (Fig. 2). In ovarian cancer samples, mitochondria were stained by all three different monoclonal antibodies, but we found a lack of staining in a considerable number of cancer cells (Fig. 3). The HSP60 immunoreactivity showed the clearest staining pattern which allowed individual mitochondria to be identified within the cancer cells, hence this antibody was chosen for the further clinical investigation.

**Fig. 4:**
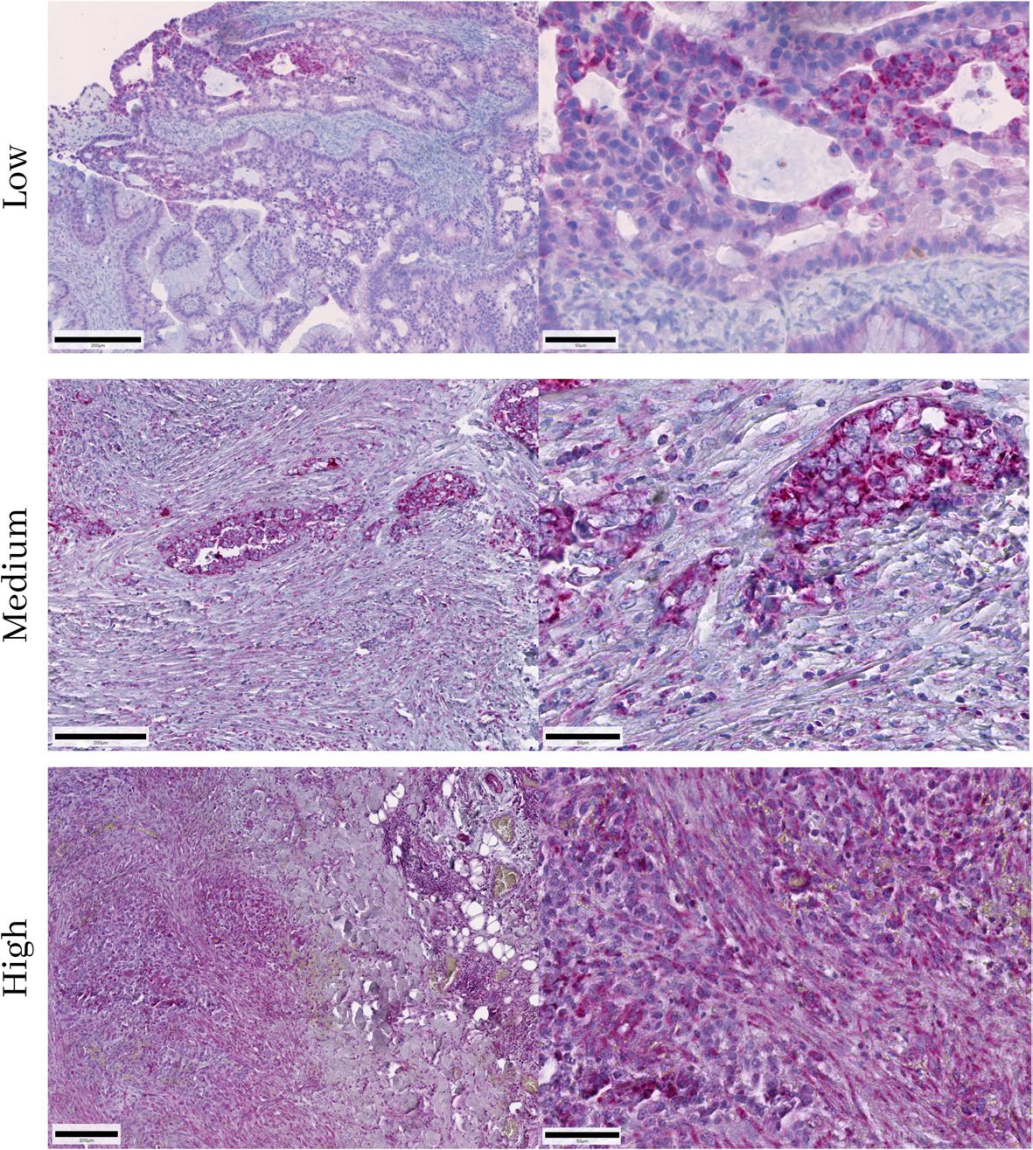
Low, intermediate, and high mitochondrial content from three different samples of ovarian cancer using the HSP-60 monoclonal antibody. The scale bar represents 200 µm on the left side (100 µm at High) and 50 µm on the right side

Next, 160 patient samples including primary ovarian cancers (*n* = 155), borderline tumours (*n* = 24) and recurrent ovarian cancers (*n* = 26) were immunohistochemically evaluated with HSP60. In total, 26 cancer patients were classified as group 1 (low mitochondrial content), 50 patients were assigned to group 2 (intermediate mitochondrial content), and 84 patients were allocated to group 3 (high mitochondrial content). Representative IHC images are shown in Fig. 4.

With regard to classical histological classification, the tumours were grouped into the following entities (see Table 3 for a summary of the results): 16 serous carcinomas, 9 serous papillary carcinomas and one mucinous carcinoma were present in group 1 with low mitochondrial content; and 23 serous papillary carcinomas, 21 serous carcinomas, two mucinous carcinomas, one papillary carcinoma and three mixed forms in group 2 with intermediate mitochondrial content. Group 3 with hight mitochondrial content was dominated by 42 serous carcinomas, followed by 35 serous papillary carcinomas, four papillary carcinomas and three mucinous carcinomas. In group 1, purely serous carcinomas were predominant, whereas in group 2 serous and serous papillary carcinomas were almost equally frequently present. Group 3 again showed the highest number of serous carcinomas. Papillary, mucinous, and mixed forms of carcinoma occurred only in small numbers in all three groups and showed no specific distribution with respect to mitochondrial content.

**Table 3:** Distribution of different mitochondrial content in histological subtypes of ovarian cancer. For statistical evaluation of the influence of the different mitochondrial content on survival see Fig. 6

	**Low mitochondrial content**	**Medium mitochondrial content**	**High mitochondrial content**
Serous	16 (62%)	23 (46%)	42 (50%)
Serous papillary	9 (35%)	21 (42%)	35 (42%)
Papillary	0 (0%)	1 (2%)	4 (5%)
Mucinous	1 (4%)	2 (4%)	3 (4%)
Mixed		3 (6%)	
Total	26 (16%)	50 (31%)	84 (53%)

**Fig. 5:**
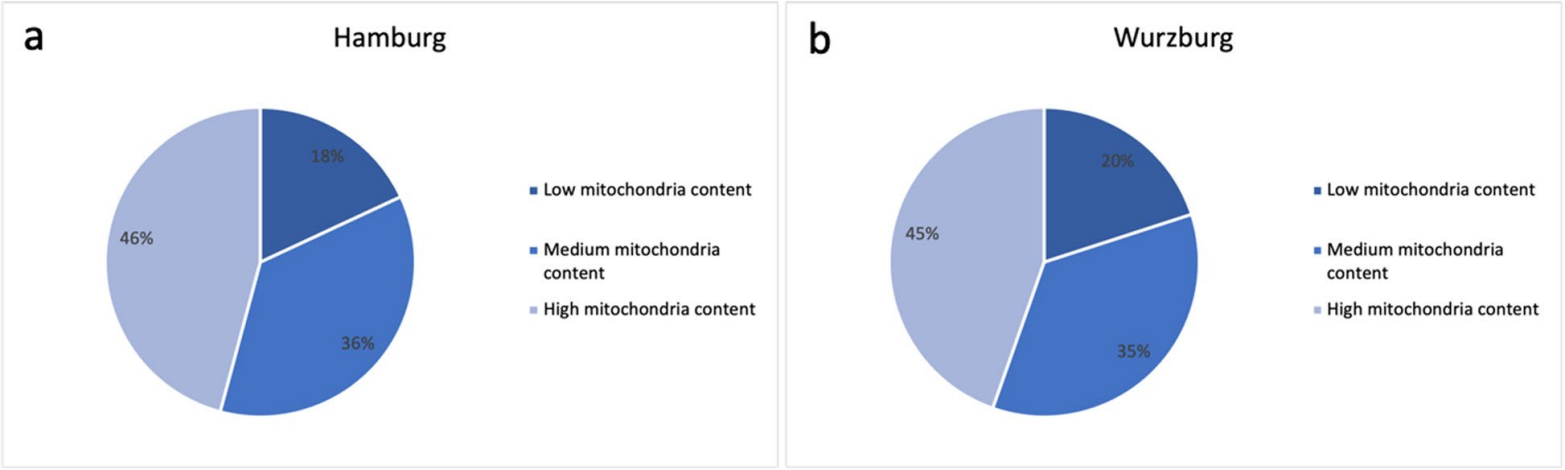
Distribution of the percentages of mitochondria of the Hamburg collective (**a**) and the Wurzburg collective (**b**). Both collectives show a comparable distribution with regard to the mitochondrial content low, medium and high

44 samples were evaluated from metastatic sites (omentum and peritoneal wall). Of these, 8 (≈18%) had low, 16 (≈36%) had medium and 20 (≈45%) had high mitochondrial content. Among all samples, we found a percentage of 16% in the low mitochondria group, 31% in the medium mitochondria group and 52% in the high mitochondria group. The results of the metastases sites are thus comparable to the overall results and did not differ statistically. To validate the sections from the Wurzburg collective which were collected between 1990 to 1997, we compared the mitochondrial content of the different collectives and found that the proportion of different mitochondrial content between the two groups was very comparable (see Fig. 5) indicating that this finding is not a single center occurrence.

### Ovarian cancers are associated with decreased overall survival as mitochondrial content increases

To assess the clinical impact of mitochondrial content on further cancer progression, correlation with histopathological and clinical parameters were studied. Only in a subgroup of the collective of ovarian cancers (*n* = 84) complete long-term clinical survival data were available. A division into three subgroups according to low (= 1), intermediate (= 2), and high (= 3) mitochondrial content was used as the basis for Kaplan–Meier analyses and log-rank testing in which the hazard ratio was determined.

**Fig. 6:**
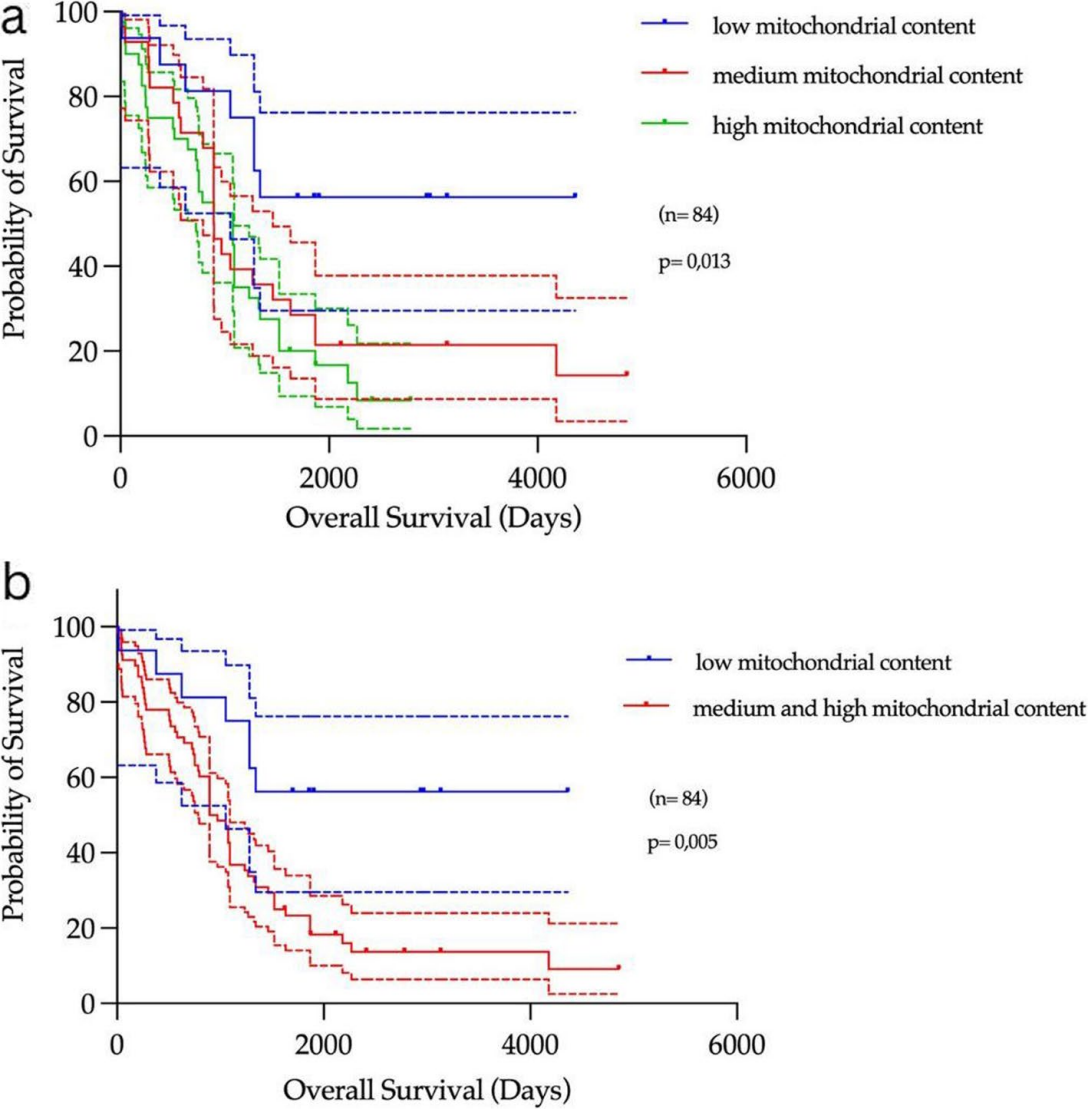
Kaplan–Meier analyses **a**) Overall survival compared in 84 cases depending on low (blue) to medium (red) and high (green) mitochondrial content. **b** Overall survival compared in 84 cases depending on low (blue) to medium and high (red) mitochondrial content. The clinical data show a clear result: Patients with tumours with low mitochondrial content survive significantly longer than those with tumours with higher mitochondrial content

**Fig. 7:**
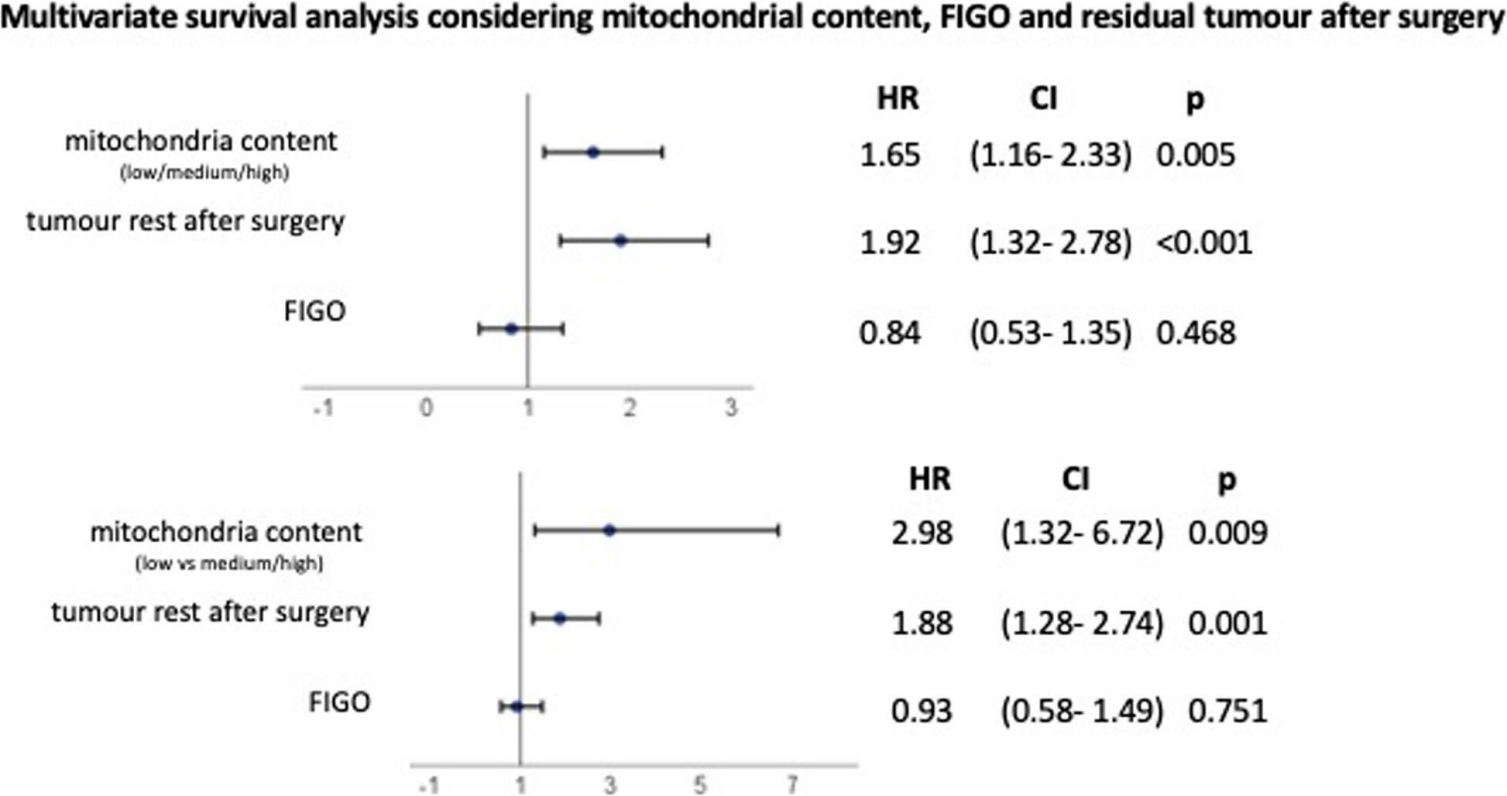
Multivariate survival analysis considering mitochondrial content, FIGO State and residual tumour after surgery. It can be seen that mitochondrial content is still significant when FIGO and tumour remnant are considered as covariates

First, the survival in years of all three groups (low, medium, high) was compared and evaluated in a Kaplan–Meier curve as shown in Fig. 6a. It can be clearly seen that patients with a tumour with few mitochondria exhibit a significantly higher and longer probability of survival. Reduced survival was recorded in patients with tumours with intermediate and high mitochondrial content. The result was statistically significant with a *p*-value of 0.013. In a second statistical analysis, the medium and high groups were merged into one high group, as shown in Fig. 6b, and contrasted with low mitochondrial content. In this analysis, the difference in survival probabilities is even more apparent. The median survival of the population containing low mitochondria could not be determined as the survival probability was always above 50%. The median survival of the population with high mitochondria content was 2.5 years. The hazard ratio (high/low) was 2.26 and the 95% confidence interval was 1.28 to 4.0, indicating that there is a correlation between high mitochondrial content and reduced probability of survival (Tables 4 and 5). To add more clinical variables to our analysis, we performed a multivariate analysis in which we added FIGO stages and tumour remnants after surgery (Fig. 7). The mitochondrial value was also significant when adding these variables.

**Table 4:** Statistical analysis of Fig. 6a using Log-rank (Mantel-Cox) test, Logrank test for trend and Gehan-Breslow-Wilcoxon test. All tests have shown statistical significance

	**Log-rank (Mantel-Cox) test (recommended)**	**Logrank test for trend (recommended)**	**Gehan-Breslow-Wilcoxon test**
Chi square	8,602	8,006	6,063
df	2	1	2
*P* value	0,0136	0,0047	0,0482
Are the survival curves sig different?	Yes	Yes	Yes

**Table 5:** Statistical analysis of Fig. 6b using Long-rank (Mantel-Cox) test and Gehan-Breslow-Wilcoxon test. All tests have shown statistical significance. In addition, the ratio and the 95% confidence interval of the ratio are given

	**Log-rank (Mantel-Cox) test (recommended)**	**Gehan-Breslow-Wilcoxon test**
Chi square	7,866	5,646
df	1	1
*P* value	0,0050	0,0175
Are the survival curves sig different?	Yes	Yes
**Hazard Ratio (Mantel–Haenszel)**	**A/B**	**B/A**
**Ratio (and its reciprocal)**	0,4431	2,257
***95% CI of ratio***	0,2509 to 0,7826	1,278 to 3,986
**Hazard Ratio (logrank)**	**A/B**	**B/A**
**Ratio (and its reciprocal)**	0,3501	2,857
**95% CI of ratio**	0,2006 to 0,6109	1,637 to 4,985

### Cells from human ovarian cancer cell lines grown as xenografts show comparable results to the clinical studies but differ if cells are grown in vitro

**Fig. 8:**
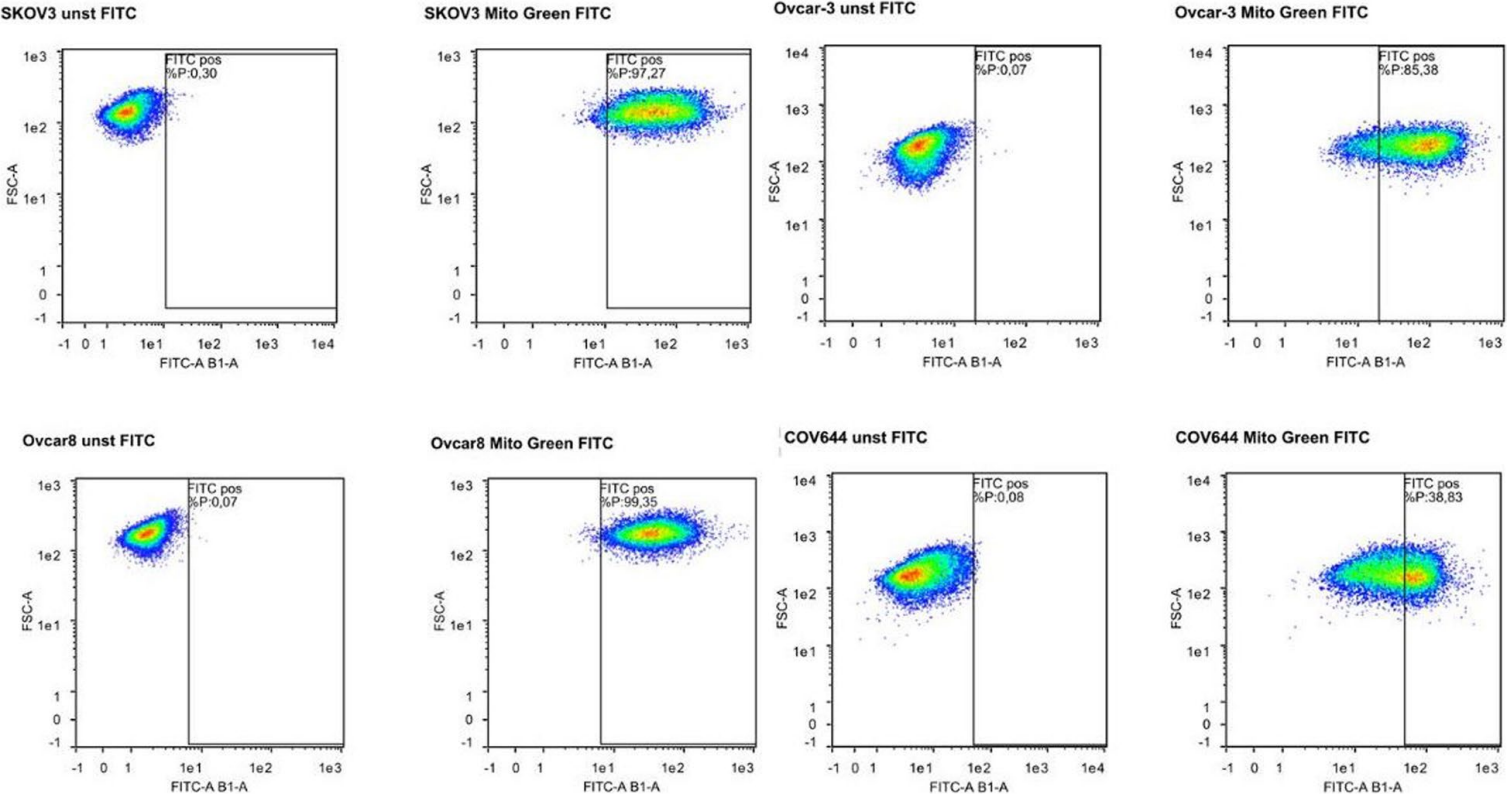
Dot-plots of four different human ovarian cancer lines (OVCAR8, SKOV3, OVCAR3 and COV644) using MitoTracker Green showing unstained and stained plots

**Fig. 9:**
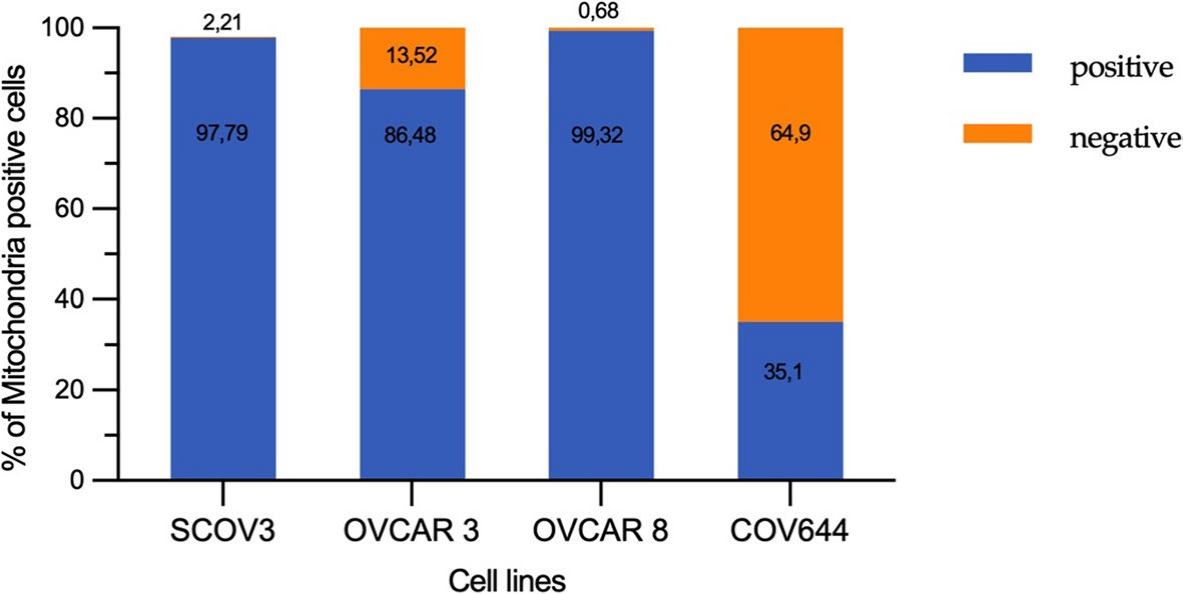
Median percentage of three independently performed tests of mitochondria positive cells of cells from four different ovarian cancer cell lines using Mitotracker green. Large differences in mitochondria positivity are seen between cell lines from a minimum of 35,1% (COV644) to a maximum of 99.3% (OVCAR8)

In flow cytometric analyses of ovarian cancer cells grown in vitro derived from four human ovarian cancer cell lines, mitochondria in living cells were detected and set in relation to live and dead cells, respectively. Overall, a large difference in the mitochondrial content between the different tumour lines can be observed. In cell lines such as OVCAR8 and SKOV3, mitochondria were detected in nearly 100% of the live cancer cells. The cell line COV644 showed the lowest mitochondrial presence with only 35% of the live cancer cells containing mitochondria (Figs. 8 and 9).

**Fig. 10:**
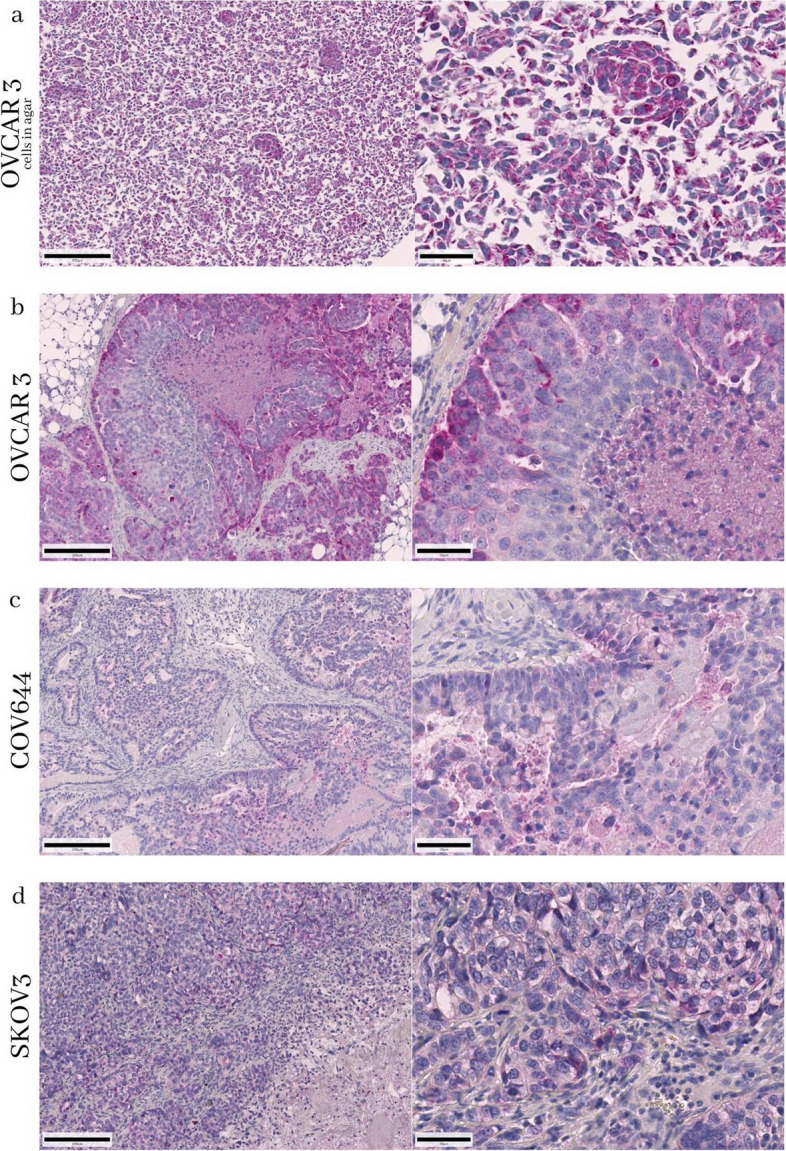
Immunohistochemistry with HSP60 in OVCAR3 cells in agar in vitro grown (**a**), OVCAR3 in vivo grown (**b**), COV644 in vivo grown (**c**) and SKOV3 in vivo grown (**d**) for comparison. The scale bar represents 200 µm on the left side and 50 µm on the right side

Next, the live cell results were correlated with immunohistochemical detection of mitochondria in in vivo grown COV644, SKOV3 and OVCAR3 xenograft ovarian cancer cells, which were formalin-fixed and paraffin-embedded (FFPE) and were thus processed in the same way as the clinical cancer tissue samples. In addition, we compared these to in vitro grown OVCAR 3 cells fixed in agar (Fig. 10a).

These results showed mitochondrial density in the low to intermediate range for COV644 cells similar to the flow cytometric assay results (Figs. 9 and 10c). In contrast, SKOV3 cells showed mainly medium to high mitochondrial content, while flow cytometry analysis showed content close to 100% (Figs. 9 and 10d). Tumours with intermediate mitochondrial density were found in both in vitro (Fig. 10a) and in vivo (Fig. 10b) OVCAR3 preparations. This agrees with the results of the flow cytometry analysis. Therefore, a good correlation was observed between the different methods of mitochondria detection in all three cell lines with different mitochondria content and the in vivo and in vitro results could be compared from the methodological point of view*.*

## Discussion

In this study, the presence of mitochondria in human ovarian cancer cells in patient samples was semi-quantitatively determined and correlated with the patients’ prognoses. In our patient cohort, which included both primary and recurrent ovarian cancer samples, a highly variable density and distribution of mitochondria were found. No significant differences were found between primary, recurrent or borderline tumours with respect to mitochondrial content. We were able to clearly determine that tumours with higher numbers of mitochondria positive cancer cells had a significantly worse clinical outcome as these patients died significantly earlier than patients whose tumours contained fewer cancer cells containing mitochondria. These patients had significantly longer survival and lower death rates. The patients with tumours with low mitochondria showed a survival probability of over 50%, while the median survival in patients with tumours with high mitochondria levels was only 2.5 years [25, 26].

Two opposing roles relevant for malignant progression including peritoneal metastasis can be attributed to mitochondria, namely, its pro-metastatic role as the provider of the energy rich substrate ATP that fosters metastasis on the one hand and the execution of a pro-apoptotic mitochondrial-based caspase pathway that limits metastasis on the other [27]. Our results clearly indicate that the energy providing function of mitochondria is of greater importance for the intraperitoneal spread of ovarian cancer cells than the execution of the apoptotic pathway by mitochondria. The results of the survival analysis suggest that the function of a suicidal weapon store is of lesser importance for metastasis formation of ovarian cancer cells than the function of energy provision for cellular activities in which a high mitochondrial content is associated with poorer survival. This observation may be explained in part by the occurrence of high interstitial fluid pressure within solid tumours, which shields the majority of cancer cells from attack by chemotherapeutic drugs because they cannot enter the mass of the primary tumour as they are too far away from circulation [28, 29]. Hence the functions of a suicidal weapon store are not needed in cancer cells further away from functioning exchange blood vessels. As the cancer cells can regulate the number of mitochondria in their cytoplasm, the low mitochondrial content does not seem to be a disadvantage for the cancer cells. Hence this switch from high to low mitochondrial numbers may explain why ovarian cancer is so difficult to treat with chemotherapy. Cancer cells with mitochondria are targeted, while those with low or no mitochondrial content are not affected by the apoptotic pathways triggered by chemotherapy.

Because of the increased glycolysis measured in cancer cells, it has been hypothesised that mitochondrial involvement plays a diminished role in malignant progression [30]. However, increased energy provision through mitochondrial pathways is present in a variety of cancers [13, 31]. Because of these significant differences of the roles of mitochondria in cancer, we hypothesised that cancer cells can dynamically regulate their mitochondrial density. As a first step, we therefore analysed mitochondrial content in human ovarian cancer cells from different ovarian cancer lines grown both in vitro and in vivo in immunodeficient mice. While in vitro grown SKOV3 cells were 97,79% and OVCAR8 cells were 99,32% positive for mitochondria, mitochondrial number dropped considerably when the same cells were grown in vivo. These results indicate that ovarian cancer cells to change their mitochondrial number according to their environment, e. g. whether they grow in 2D in vitro or in 3D in vivo and thus indicates that they adopt to their metabolic and survival needs. This in turn may have consequences in for the success of chemotherapy as it has been described in oesophageal cancer cells where low mitochondrial DNA number induces chemotherapy resistance via the induction of EMT [32]. Cells with initially low mitochondrial content are spared during a course of chemotherapy and can restore high mitochondrial number once chemotherapy has stopped and can thus grow more aggressively again. Strong metabolic heterogeneity and plasticity has also been found in other tumours, for example, lung carcinomas [31, 33]. This heterogeneity within tumour cells poses an enormous problem for therapeutic options, as molecularly targeted therapies cannot be effective [34].

The mitochondrial respiratory chain is primarily used for energy provision, particularly in quiescent cancer cells in poorly vascularised or non-vascularised regions [35]. As the chemotherapeutic agents act on the rapidly proliferating cells, the cancer cells that perform their ATP production via OXPHOS are associated with increased resistance to chemotherapeutic agents, whereas cancer cells that follow the Warburg hypothesis of typical glycolysis are more sensitive to chemotherapeutic agents [19, 36]. Wang et al. found increased mtDNA copy numbers in ovarian tumour cells [37] and Lim et al. demonstrated that intact mitochondria exhibited high oxidative phosphorylation activity by isolating them from ovarian tumour cells [38]. These observations have also been made in leukaemia and melanoma cancer cells [39, 40]. Increased energy provision by OXPHOS results in increased IL-6 production, leading to the longer survival and increased proliferation of cancer cells [41, 42]. In addition, chemotherapy efficacy and recurrence-free survival are decreased [43].

The increased level of active mitochondria in ovarian cancer cells leads to increased OXPHOS activity and thereby increased sensitivity to inhibitors of the OXPHOS system [6, 44]. Our hypothesis that the poorer clinical outcome we describe is related to increased mitochondrial function can be confirmed.

### Limitations

Although the relatively small sample size revealed statistically very significant results, out study should be confirmed by a prospective one.

## Conclusion

Mitochondria primarily act as energy providers in metastasising ovarian cancer cells, while their function as inducers of apoptosis is of lesser importance. The ability of ovarian cancer cells to regulate their mitochondrial content may also add to their resistance to chemotherapy as low mitochondrial content is associated with chemotherapy resistance [32].

## Data Availability

The data that support the findings of this study are available from the corresponding author upon reasonable request.
